# Identifying Work-Related Injuries Among Healthcare Workers in Makkah Hospitals, Saudi Arabia: A Cross-Sectional Study in 2024

**DOI:** 10.7759/cureus.67323

**Published:** 2024-08-20

**Authors:** Maria A Alqithami, Amani ALsadi, Mohammed Tashkandi, Rehab Gaafar

**Affiliations:** 1 Preventive Medicine, Preventive Medicine Center in Makkah, Makkah, SAU; 2 Preventive Medicine, Ministry of Health, Makkah, SAU; 3 Preventive Medicine, King Abdullah Medical City, Makkah, SAU

**Keywords:** occupational safety, needle stick and sharp injury, accidental injury, work-related injury, hazards, injuries, healthcare workers

## Abstract

Background

Work-related injuries (WRIs) are a major occupational health issue among healthcare workers (HCWs) worldwide. HCWs face numerous daily hazards including needlestick injuries, chemical exposures, ergonomic strains, and psychological stressors crucial for their health and healthcare system functionality. In Makkah, Saudi Arabia, healthcare infrastructure advances raise concerns about work-related injuries among HCWs. This study in Makkah hospitals aims to identify, understand, and manage WRIs for improved occupational health guidelines and strategies.

Methods

This descriptive cross-sectional study on HCWs was conducted at Makkah hospitals using an electronic questionnaire that investigated the demographics, work-related injuries, and occupational hazards. The data collected from the retrieved questionnaires were analyzed using the IBM SPSS Statistics for Windows, Version 26.0 (Released 2019; IBM Corp., Armonk, New York, United States).

Results

Among 379 enrolled HCWs, 172 (49.3%) were physicians and 89 (19.8%) were nurses; 304 (80.2%) of the total participants knew about occupational safety. The total incidence of WRIs was 67.8%. WRIs were significantly associated with age (P˂0.001), gender (P=0.02), educational level (P˂0.001), profession (P˂0.001), working hours (P˂0.001), and shift time (P˂0.001).

Conclusion

WRIs were highly prevalent among HCWs with varying rates based on the type of injury and the frequency of injury. WRIs were associated with various factors including age, gender, education, profession, working house, and shift time of the participants.

## Introduction

Healthcare workers (HCWs) encompass direct care providers such as physicians, pharmacists, laboratory technicians, and nurses, as well as indirect providers like healthcare administrators [1]. The safety and well-being of HCWs are critical not only for their individual health but also for the sustained functionality of healthcare systems worldwide. HCWs face various health hazards at work, including biological, chemical, physical, and psychological hazards, affecting up to 50% of them [1]. The United States Occupational Health and Safety Administration (OSHA) estimated that 5.6 million HCWs are at risk of occupational exposure to different blood-borne pathogens due to needlestick injuries [2]. Globally, an estimated 32.4-44.5% of HCWs report at least one event of accidental needle-stick injury (NSI) or sharp injury each year [3,4]. A study done in a few governmental hospitals in the Kingdom of Saudi Arabia (KSA) estimated that the annual NSI incidence was 3.2 per 100 occupied beds, and nurses were the most affected job category [5]. A recent study conducted in a hospital in the Medina region estimated the annual incidence of NSIs among healthcare personnel at 32% [6]. Another study reported that 29.8% of the dental assistants working in private dental clinics in Jeddah, KSA, experienced at least one event of NSI since starting their career [7]. HCWs are at risk of getting infected with blood-borne pathogens through NSIs at work. Globally, about 40% of HBV and HCV, and 2.5% of HIV/AIDS cases among HCWs are due to NSIs and more than 90% of these infections occur in healthcare settings in low-income countries where adherence to standard precautions is poor [8]. Despite the high incidence and risk of adverse health consequences of NSIs [9], there is a marked underreporting of NSI incidents by HCWs [7,10,11]. A study in private clinics in Jeddah, KSA found that more than half (63%) of the dental HCWs experiencing NSIs do not report their injuries to the authorities [7].

In recent years, the KSA, particularly the city of Makkah, has witnessed significant developments in healthcare infrastructure and service delivery. However, alongside these advancements, concerns persist regarding the frequency and nature of WRIs among HCWs in Makkah hospitals. Understanding the types, prevalence, and circumstances of WRIs is fundamental to enhancing preventive measures and ensuring effective management strategies tailored to local healthcare settings. This study aims to delineate the spectrum of work-related injuries and occupational risk issues among HCWs in Makkah hospitals. The identification of these incidents is crucial for refining existing guidelines and implementing improved management strategies. 

## Materials and methods

This was a descriptive cross-sectional study conducted at hospitals in Makkah including Al-Noor Specialist Hospital, Hera General Hospital, King Abdullah Medical City, King Abdulaziz Hospital, King Faisal Hospital, and Security Forces Hospital, from February to June, 2024, focusing on HCWs aged 18-60 years. The study included a range of professionals such as physicians, nurses, pharmacists, radiology staff, laboratory specialists, dental staff, technicians, and allied medical professionals. The study was approved by the King Abdullah Medical City Institutional Review Board (approval number: 24-1206).

Inclusion and exclusion criteria

The inclusion criteria for the study specified that only individuals currently employed as HCWs in selected hospitals in Makkah were eligible to participate. To ensure adequate exposure to the work environment, participants were required to have been employed at the hospital for at least six months. Exclusion criteria were set to omit non-HCWs, such as administrative staff and visitors, as well as temporary or short-term employees who had been employed for less than six months, to eliminate potential bias due to limited exposure. Interns and health volunteers were also excluded from the study.

Sample calculation and selection

According to 2023 data from the Ministry of Health (MoH), the total HCW population in Makkah was reported as 24,704. Using Raosoft software with a 95% confidence level and a 5% margin of error, the study determined a minimum sample size of 379 HCWs from the total population of 24,704 in Makkah. Participants were selected using a multistage sampling technique. Initially, a list of all hospitals in Makkah was obtained, and a random sample of hospitals was selected. Within each selected hospital, departments were stratified based on the nature of healthcare services provided, and a random sample of departments was chosen to capture a wide range of HCW experiences and exposure to potential work-related injuries. Finally, within each selected department, HCWs were stratified by professions, such as doctors, nurses, technicians, and support staff, and a random sample of HCWs from each professional category was invited to participate in the study.

Data collection

Data were collected through an electronic, validated questionnaire that had been previously utilized in comparable studies [12]. The questionnaire consisted of three sections: Section A focused on sociodemographic characteristics, including gender, age, occupational category, hospital affiliation, nationality, marital status, profession, years of experience, medical history, availability of personal protective equipment (PPE), and work-related injuries (WRIs). Section B addressed specific questions regarding exposure to WRIs, including physical, chemical, and biological incidents, while Section C covered inquiries related to various occupational hazards encompassing biological, physical, chemical, and psychosocial injuries.

Data analysis

Data were entered and analyzed using IBM SPSS Statistics for Windows, Version 26.0 (Released 2019; IBM Corp., Armonk, New York, United States). Descriptive statistics, including frequencies and percentages, were used to summarize qualitative variables, while means and standard deviations (SD) were employed to describe quantitative variables. The Chi-square test was utilized to compare qualitative variables according to sex, with a p-value <0.05 considered statistically significant.

## Results

Among the included 379 HCWs, 123 (32.5%) belonged to the age group of 26-30 years. Females (n=194, 51.2%) were more compared to males (n=185, 48.8%). More than one-half of the subjects were married (n=250, 65.9%) and most of the participants were Saudi (n=265, 69.9%). The largest proportion of the participants had a bachelor‘s degree (n=218, 57.5%), followed by those who completed Board (n=112, 29.6%). Around one-third of the participants (n=136, 35.9%) had an experience of more than 10 years. Physicians represented almost one-half of the sample (n=172, 45.4%), followed by nurses (n=89, 23.5%). Most of the participants had no chronic diseases (n=288, 76.0%). The largest proportion of the participants reported working at KAMC (n=108, 28.5%), followed by Hera General Hospital (n=60, 15.8%). Almost one-half of the HCWs reported working for eight hours daily (n=188, 49.6%) and one-half reported mixed shifts (n=167, 46.4%). The majority knew about occupational safety and the protocols of health administration of NSI (Table 1).

**Table 1:** Demographic characteristics of study participants (N=379) NSI: needle-stick injury; OSHA: Occupational Health and Safety Administration

Characteristics		Frequency	Percentage
Age group, years	18–25	5	1.3%
	26–30	123	32.5%
	31–35	104	27.4%
	36–40	84	22.2%
	41–45	21	5.5%
	46–50	19	5%
	>51	23	6.1%
Gender	Female	194	51.2%
	Male	185	48.8%
Marital status	Divorced	14	3.7%
	Married	250	65.9%
	Single	115	30.4%
Nationality	Non-Saudi	114	30.1%
	Saudi	265	69.9%
Educational level	Bachelor's	218	57.5%
	Board	112	29.6%
	Diploma	29	7.7%
	Fellowship	20	5.2%
Work experience	Between 1-4 years	101	26.6%
	Between 5-10 years	131	34.6%
	Less than 1 year	11	2.9%
	More than >10 years	136	35.9%
Profession	Dentist	15	3.9%
	Laboratory staff	27	7.2%
	Nurse	89	23.5%
	Pharmacist	16	4.2%
	Physician	172	45.4%
	Radiology staff	16	4.2%
	Technician	44	11.6%
Chronic diseases	No	288	76.0%
	Yes	91	24.0%
Workplace	Al-Noor Specialist Hospital	49	12.9%
	Hera General Hospital	60	15.8%
	King Abdullah Medical City	108	28.5%
	King Abdulaziz Hospital	43	11.4%
	King Faisal Hospital	49	12.9%
	Security Forces Hospital	40	10.6%
	Other	30	7.9%
Hours of working	8	188	49.6%
	8-12	167	44.1%
	>12	24	6.3%
Work shift	Evening	13	3.4%
	Mixed	176	46.4%
	Morning	190	50.2%
Knowledge of OSHA NSI protocols	No	75	19.8%
	Yes	304	80.2%

The organizational factors of the HCWs are shown in Table 2. The largest proportion of the workers reported that PPE and sharps containers are always available (n=274, 72.3%, and n=318, 83.9%, respectively). More than one-half reported always using PPE as per protocol (n=202, 53.3%).

**Table 2:** Organizational factors

Organizational factors	Always		Never		Rarely		Sometimes		Usually	
	Frequency	Percentage	Frequency	Percentage	Frequency	Percentage	Frequency	Percentage	Frequency	Percentage
Personal protective equipment availability	274	72.3%	1	0.3%	4	1.1%	30	7.9%	70	18.5%
Sharps container availability	318	83.9%	0	0.0%	15	3.9%	4	1.1%	42	11.1%
Use of personal protective equipment as per protocol	202	53.3%	10	2.6%	26	6.9%	65	17.2%	76	20.1%

WRIs were assessed through 10 questions which are displayed in Table 3 with the distribution of replies. The assessment of WRIs during the previous six months revealed that most of the participants reported that they never experienced NSIs (n=332, 87.6%), injuries from contaminated objects (n=331, 87.3%), eye/mouth splashes (n=333, 87.9%), cuts with sharp objects (n=331, 82%), any work-related skin rashes (n=313, 84.0%) or injuries due to falls (n=302, 79.7%). Less than one-half reported that they never experienced back injuries or pain (n=161, 42.5%), whereas 83 (22%) reported that they experienced it six times or more during the previous six months. Most of the HCWs stated that they never experienced burn injuries (n=362, 95.5%), poisoning symptoms (n=354, 93.4%), or electrical shock (n=370, 97.6%).

**Table 3:** Work-related injuries experienced by the healthcare workers in the hospital setting (N=379) NSI: needle stick injuries

	Never		1 time		2 times		3 times		4 times		5 times		6 times and more	
	Frequency	Percentage	Frequency	Percentage	Frequency	Percentage	Frequency	Percentage	Frequency	Percentage	Frequency	Percentage	Frequency	Percentage
Have you experienced NSIs at your workplace in the last six months	332	87.6%	23	6.1%	13	3.4%	6	1.6%	5	1.3%	0	0.0%	0	0.0%
Have you experienced injuries from contaminated objects at your workplace in the last six months	331	87.3%	26	6.9%	17	4.5%	5	1.3%	0	0.0%	0	0.0%	0	0.0%
Have you experienced back injuries/pain at your workplace in the last six months	161	42.5%	26	6.9%	53	14%	16	4.2%	28	7.4%	12	3.2%	83	22%
Have you experienced eye/mouth splashes at your workplace in the last six months	333	87.9%	19	5.0%	17	4.5%	8	2.1%	2	0.5%	0	0.0%	0	0.0%
Have you experienced cuts with sharp objects at your workplace in the last six months	311	82.1%	35	9.2%	27	7.1%	3	0.8%	2	0.5%	1	0.3%	0	0.0%
Have you encountered any work-related skin rashes in the past six months	313	84.0%	35	9.4%	9	2.4%	8	2.1%	7	1.6%	7	1.8%	0	0.0%
Have you experienced injuries due to falls at your workplace in the last six months	302	79.7%	62	16.4%	6	1.6%	2	0.5%	2	0.5%	2	0.5%	3	0.8%
Have you experienced burn injuries at your workplace in the last six months?	362	95.5%	12	3.2%	2	0.5%	3	0.8%	0	0.0%	0	0.0%	0	0.0%
Have you experienced poisoning symptoms related to your workplace in the last six months	354	93.4%	11	2.9%	9	2.4%	2	0.5%	3	0.8%	0	0.0%	0	0.0%
Have you experienced electrical shocks at your workplace in the last six months	370	97.6%	8	2.1%	1	0.3%	0	0.0%	0	0.0%	0	0.0%	0	0.0%

A total of 11 questions were used to identify the occupational hazards (Table 4). The largest proportion of the subjects stated that they always have direct contact with patients (n=228, 60.2%) and the patient's blood and body fluids (n=117, 30.9%). The largest proportions reported that they never have direct contact with chemicals (n=165, 43.5%), or radiation (n=194, 51.2%). A total of 118 (31.1%) participants reported that they sometimes worked in an environment of loud noise. However, the largest proportion of the workers stated that they never work in an environment with high-temperature variation (n=131, 34.6%), lack of space (n=132, 34.8%), poor air quality (n=202, 53.3%), poor lighting (n=227, 59.9%), risk of falls (n=198, 52.2%), or electrical hazards (n=228, 60.2%).

**Table 4:** Occupational hazards faced by the healthcare workers (N=379)

Questions	Always		Often		Sometimes		Rarely		Never	
	Frequency	Percentage	Frequency	Percentage	Frequency	Percentage	Frequency	Percentage	Frequency	Percentage
Do you have direct contact with patients	228	60.2%	32	8.4%	70	18.5%	16	4.2%	33	8.7%
Do you have direct contact with chemical products	37	9.8%	26	6.9%	73	19.3%	78	20.6%	165	43.5%
Do you have direct contact with radiation	40	10.6%	21	5.5%	63	16.6 %	61	16.1%	194	51.2%
Do you have direct contact with the patient blood and body fluids or tissues	117	30.9%	31	8.2%	91	24.0%	55	14.5%	85	22.4%
Do you work in an environment where there is high-temperature variation highly cold or hot temperature	68	17.9%	56	14.8%	72	19%	52	13.7%	131	34.6%
Do you work in an environment where there is a loud noise	56	14.8%	40	10.6%	118	31.1%	82	21.6%	83	21.9%
Do you work in an environment where there is a lack of space?	34	8.9%	42	11.1%	85	22.4%	86	22.7%	132	34.8%
Do you work in an environment where there is poor air quality with little or no ventilation	16	4.2%	16	4.2%	66	17.4%	79	20.8%	202	53.3%
Do you work in an environment where there is poor lighting?	13	3.4%	20	5.3%	36	9.5%	83	21.9%	227	59.9%
Do you work in an environment where there is a risk of falls or trips?	0	0.0%	10	2.7%	61	16.1%	110	29.0%	198	52.2%
Do you work in an environment where there are electrical hazards	7	1.9%	6	1.6%	32	8.4%	106	27.9%	228	60.2%

A Chi-square test was used to compare work injuries with worker characteristics. Results revealed that work injuries had a significant association with age groups (P˂0.001), gender (P=0.02), educational level (P˂0.001), profession (P˂0.001), working hours (P˂0.001), and shift time (P˂0.001) (Table 5).

**Table 5:** Association of sociodemographic variables with the work injuries in the study participants (N=379)

Variables		Work Injuries				P value
		No		Yes		
		Frequency	Percentage	Frequency	Percentage	
Age group, years	<26	4	80.0%	1	20.0%	<0.001
	26–30	36	29.3%	87	70.7%	
	31–35	44	42.3%	60	57.7%	
	36–40	36	42.9%	48	57.1%	
	41–45	8	38.1%	13	61.9%	
	46–50	3	15.8%	16	84.2%	
	>51	20	87.0%	3	13.0%	
Gender	Female	72	37.1%	122	62.9%	0.025
	Male	95	51.4%	90	48.6%	
Marital status	Divorced	7	50.0%	7	50.0%	0.158
	Married	89	33.6%	161	66.4%	
	Single	31	27.0%	84	73.0%	
Nationality	Saudi	172	65.5%	93	34.5%	0.111
	Non-Saudi	34	25.5%	80	74.5%	
Educational level	Bachelor's	37	17%	181	83%	<0.001
	Board	66	59.8%	46	40.2%	
	Diploma	8	27.6%	21	72.4%	
	Fellowship	14	70%	6	30%	
Work experience	Less than 1 year	0	0.0%	11	100.0%	0.054
	Between 1-4 years	35	34.7%	66	65.3%	
	Between 5-10 years	53	40.5%	78	59.5%	
	More than >10 years	39	28.7%	97	71.3%	
Profession	Dentist	8	50.0%	8	50.0%	<0.001
	Laboratory staff	13	50.0%	13	50.0%	
	Nurse	32	36.0%	57	64.0%	
	Pharmacist	15	93.8%	1	6.3%	
	Physician	78	45.3%	94	54.7%	
	Radiology staff	14	87.5%	2	15.5%	
	Technician	39	88.6%	5	11.4%	
Chronic diseases	No	105	36.5%	183	63.5%	0.06
	Yes	24	26.4%	67	73.6%	
Hours of working	8	17	70.8%	7	29.2%	<0.001
	8-12	82	43.6%	106	56.4%	
	>12	28	16.8%	139	83.2%	
Work shift	Mixed	36	20.5%	140	79.5%	<0.001
	Morning	82	43.2%	108	56.8%	
	Evening	9	69.2%	4	30.8%	

Additionally, the analysis showed a significant association between work injuries and the knowledge of occupational safety (P˂0.001), where the highest proportion of those who reported work injuries also had knowledge (Table 6).

**Table 6:** Relation of work-relaed injuries to knowledge in the study sample (N=379) NSI: needle-stick injury

		Work Injuries				P value
		No		Yes		
		Frequency	Percentage	Frequency	Percentage	
Knowledge of the Occupational Safety and Health Administration NSI protocols	No	9	7.4%	56	21.8%	<0.001
	Yes	113	92.6%	201	78.2%	

## Discussion

This study assessed the types of WRIs and ill health among HCWs in hospitals in Makkah, Saudi Arabia. HCWs include those who provide direct care such as physicians, pharmacists, nurses, and laboratory technicians, and those who provide indirect service such as healthcare administrators. In this study, physicians were the predominant profession, followed by nurses. Additionally, there was a Saudi predominance as Saudis represented more than one-half of the subjects. 

Among the total HCWs, we found that the majority of them knew about occupational safety and protocols for needle sticks. In contrast to our findings, in an Ethiopian study that included 213 nurses, it was found that the knowledge of standard precautions was adequate among only 35.64% [13]. Such findings refer to a lower level of knowledge regarding the safety and protocols of NSIs among the nurses.

Regarding work conditions, a high proportion of the HCWs reported that PPE and sharps containers are always available, but a lower proportion reported using PPE as per protocol. Our findings were better than those reported in a previous Saudi study, where more than two-half of HCWs (n=274, 72.3%) reported that PPE is always available, and a higher proportion (n=318, 83.9%) reported that sharps containers are always available [14]. WRIs are self-reported injuries from incidental events, including NSIs, wounds, eye or mouth contact with contaminated substances, splashes to the face, falls, burns, back injuries, skin rashes, mechanical injuries, electrical shocks, and other types of injuries [12].

In this study, such injuries were assessed for the previous six months of conducting the study. The overall incidence of WRIs as reported by the participants was high (n=257, 67.8%). The major reported WRIs, included back injuries and pain, injuries from contaminated objects, cuts with sharp objects, and NSIs. A lower incidence was reported in a previous Saudi study, where the overall incidence of WRIs was 52% [14]. Additionally, back injuries were the major reported injuries, similar to our study, followed by eye/mouth splash injuries and then needle sticks.

A study from Ghana reported incidence was 1.63 injuries per person-year. Leading mechanisms were NSIs (35.4% of injuries), cuts from sharp objects (34.6%) [15], and a study from Iran that enrolled 802 HCWs demonstrated that 25.3% experienced NSIs at least once in the previous year [16]. In this study, NSIs were experienced one time by 23 (6.1%) participants in the previous six months, with a total of 12.4% (n=47) who reported NSI but in various frequencies over the previous six months. An HSW study from Indonesia revealed that cutting with sharp objectives, needle sticks, and falling from height were never experienced as reported by 49.6%, 52.3%, and 97.6%, respectively [17]. Higher proportions in our study reported never experiencing NSIs (n=332, 87.6%) or cutting (n=311, 82.1%), but a lower proportion compared to the previous study [17] reported never experiencing falls (86.5%).

WRIs in the current study were found to be correlated with age, gender, education, profession, working hours, and shifts. A previous study from Jeddah conducted on 387 HCWs reported correlations between occupational hazards and years of experience, profession, and working shift. Further analysis displayed that low educational levels and years of experience were predictors of occupational hazards. Those with a lower level of education and lower experience had higher odds of experiencing hazards [14]. However, work experience displayed no significant correlation with WRIs which contrasted with the previous study. Additionally, multivariate analysis wasn’t established in this study, and we couldn’t determine the predictors of WRIs.

Correlations between injuries and occupational accidents were found with gender and work experience (P=0.01). A study from Turkey showed that injury incidences were significantly higher in nurses compared to other HCWs [18]. In our study too, the highest proportion of injuries were experienced by nurses (64.0%). However, a larger frequency of physicians reported injuries. A study from Singapore displayed that the highest incidence of injuries was found among doctors (43.7%) and nurses (37.7%) [19].

Experiencing WRIs displayed significant correlations with gender, but there was no correlation with marital status or working hours as per a previous study [20]. Similarly, we found correlations between WRIs and gender as females significantly tended to experience injuries. Also, in agreement with the previous study, marital status displayed no correlations with injuries; however, in contrast to the previous study, there was a significant correlation between working hours and injuries, where working longer hours was significantly associated with experiencing injuries.

Based on a previous Saudi study, HCWs aged 26-30 years were 2.5 times more prone to experience NSIs compared to other age groups [21]. In the current study, HCWs aged 26-30 were more predisposed to experience WRIs and this agreed with the previous study. A study from Iran found that single HCWs had a higher risk of NSIs compared to married HCWs. Also, night shift work, higher educational degrees, working overtime, older age, and needle recapping were identified as significant associated factors [16]. The contrary was found in this study regarding educational degree, age, and shift as those with younger age, lower educational degrees and those working mixed shifts tended to experience injuries, whereas the results were similar regarding longer working hours.

Evidence is growing that long work hours and shift work are linked to errors in the delivery of patient care, and our findings indicate that while longer hours were associated with a higher rate of injuries, it was the mixed shifts, rather than evening or morning shifts, that were specifically linked to increased injury exposure. One study demonstrated working in a job with extended hours per week was associated with a 23% higher injury hazard rate, working in a job with overtime was associated with a 61% higher injury hazard rate, and working in a job with any overtime or extended hours schedule was associated with a 38% higher injury hazard rate [20]. HCWs face several health hazards at work, including chemical biological, physical, and psychological hazards. A cross-sectional study was conducted in eight major health facilities in Kampala to estimate the occupational health hazards faced by HCWs and their measures. According to the results, 50% experience health hazards [22]. 

A previous study of HCWs revealed that physical hazards were the main injuries reported (32.9%), followed by chemical (28%) and biological hazards (17.1%) [23]. In the current study, the largest proportion reported direct contact with the patients, and this may lead to physical injuries, followed by contact with blood, body fluids, and tissues of the patients.

This study has some limitations, including its cross-sectional design, which could not identify a causal effect. Additionally, the reliance on questionnaires introduces potential recall bias and subjective observations, which could impact the generalizability of the findings.

## Conclusions

WRIs were highly prevalent among HCWs with varying rates based on the type of injury and the frequency of injury. Despite the availability of PPE, a low proportion of HCWs used it following the protocol. WRIs were associated with age, gender, education, profession, working house, and shift time of the participants. WRIs were associated with younger age, females, lower education, working longer hours, mixed shifts, nurses, physician, dentist and laboratory staff.
